# Ocular sequelae of epidermal necrolysis: French national audit of practices, literature review and proposed management

**DOI:** 10.1186/s13023-023-02616-6

**Published:** 2023-03-11

**Authors:** Dhyna Thorel, Saskia Ingen-Housz-Oro, Daniel Benaïm, Vincent Daien, Eric Gabison, Valentine Saunier, Laurence Béral, David Touboul, Dominique Brémond-Gignac, Matthieu Robert, Robin Vasseur, Gérard Royer, Olivier Dereure, Brigitte Milpied, Claire Bernier, Anne Welfringer-Morin, Christine Bodemer, Nadège Cordel, Marie Tauber, Carole Burillon, Marion Servant, Chloe Couret, Bertrand Vabres, Florence Tétart, Myriam Cassagne, Marie-Ange Kuoch, Marc Muraine, Agnès Delcampe, Julie Gueudry

**Affiliations:** 1 Ophthalmology Department, CHU Charles Nicolle Rouen, Rouen, France; 2 Reference Center for Toxic Bullous Dermatoses and Severe Drug Reactions TOXIBUL, Créteil, France; 3 Dermatology Department, AP-HP, Henri Mondor Hospital, Univ Paris Est Créteil EpidermE, Créteil, France; 4 Ophthalmology Department, AP-HP, Henri Mondor Hospital, Créteil, France; 5 Ophthalmology Department CHU Montpellier, Montpellier, France; 6 6Ophthalmology Department, Hôpital Fondation Adolphe de Rothschild, Paris, France; 7 Ophthalmology Department, CHU Bordeaux, Bordeaux, France; 8 Ophthalmology Department, CHU Pointe À Pitre, Pointe À Pitre, Guadeloupe, France; 9 Ophthalmology Department, Hôpital Universitaire Necker Enfants-Malades, APHP, Paris, France; 10 Ophthalmology Department, CHU de Nantes, Nantes, France; 11 Dermatology Department, CHU Montpellier, Montpellier, France; 12 Dermatology Department, CHU Bordeaux, Bordeaux, France; 13 Dermatology Department, CHU Nantes, Nantes, France; 14 Dermatology Department, AP-HP, Hôpital Universitaire Necker Enfants-Malades, APHP, Paris, France; 15 Dermatology and Clinical Immunology Department, CHU Guadeloupe, Pointe À Pitre, , Guadeloupe, France; 16 Dermatology Department, CHU de Toulouse, Toulouse, France; 17 Ophthalmolgy Department, CHU Lyon, Lyon, France; 18 Dermatology Department, CHU Charles Nicolle Rouen, Rouen, France; 19 Ophthalmology Department, CHU de Toulouse, Toulouse, France

**Keywords:** Stevens-Johnson syndrome, Toxic epidermal necrolysis, Management, Ocular involvement, Treatment, Drug reaction, Eye, Sequelae

## Abstract

Stevens-Johnson Syndrome (SJS) and toxic epidermal necrolysis (TEN) are serious and rare diseases, most often drug-induced, and their incidence has been estimated at 6 cases/million/year in France. SJS and TEN belong to the same spectrum of disease known as epidermal necrolysis (EN). They are characterized by more or less extensive epidermal detachment, associated with mucous membrane involvement, and may be complicated during the acute phase by fatal multiorgan failure. SJS and TEN can lead to severe ophthalmologic sequelae. There are no recommendations for ocular management during the chronic phase. We conducted a national audit of current practice in the 11 sites of the French reference center for toxic bullous dermatoses and a review of the literature to establish therapeutic consensus guidelines. Ophthalmologists and dermatologists from the French reference center for epidermal necrolysis were asked to complete a questionnaire on management practices in the chronic phase of SJS/TEN. The survey focused on the presence of a referent ophthalmologist at the center, the use of local treatments (artificial tears, corticosteroid eye drops, antibiotic-corticosteroids, antiseptics, vitamin A ointment (VA), cyclosporine, tacrolimus), the management of trichiatic eyelashes, meibomian dysfunction, symblepharons, and corneal neovascularization, as well as the contactologic solutions implemented. Eleven ophthalmologists and 9 dermatologists from 9 of the 11 centers responded to the questionnaire. Based on questionnaire results, 10/11 ophthalmologists systematically prescribed preservative-free artificial tears, and 11/11 administered VA. Antiseptic or antibiotic eye drops or antibiotic-corticosteroid eye drops were recommended as needed by 8/11 and 7/11 ophthalmologists, respectively. In case of chronic inflammation, topical cyclosporine was consistently proposed by 11/11 ophthalmologists. The removal of trichiatic eyelashes was mainly performed by 10/11 ophthalmologists. Patients were referred to a reference center for fitting of scleral lenses (10/10,100%). Based on this practice audit and literature review, we propose an evaluation form to facilitate ophthalmic data collection in the chronic phase of EN and we also propose an algorithm for the ophthalmologic management of ocular sequelae.

## Background

Epidermal necrolysis (EN), including Stevens-Johnson syndrome (SJS) and toxic epidermal necrolysis (TEN), is a severe and rare drug reaction, the incidence of which has recently been estimated at 6 cases/million/year in France [1]. EN is characterized by necrosis of the epidermis and mucosa. SJS and TEN differ in the percentage of detached-detachable body surface area (SJS < 10% and TEN ≥ 30%)[2]. Twenty to 79% of patients with acute forms of the disease have vision-threatening ocular damage [3]. The main risk factor for ocular sequelae is the severity of the initial ocular damage [4]. In addition, we recently reported that phototypes V and VI were also risk factors for greater severity of ocular sequelae [5].

The most disabling long-term sequelae of EN is ocular damage with severe visual impairment. The incidence is between 35 and 50% depending on the series [6]. After the acute phase of EN, the ocular surface remains subject to chronic inflammation that can lead to limbal stem cell deficiency, keratinization of the palpebral margin, and corneal opacification which may lead to blindness. The aim of EN management in the acute phase is to limit and prevent chronic eye disease and the blindness it can cause [6]. The mechanism of chronic ocular damage is based on a combination of physiological and mechanical aggressions of the ocular surface. Therefore, the objective of management is to limit ocular inflammation and the factors aggravating it as well as the toxicity of the treatments [6].

To date, there is no consensus on the ophthalmologic management of the ocular sequelae of EN, nor is there a standardized consensual ophthalmologic evaluation form, as we proposed in a previous study on the management of ocular involvement in the acute phase [7]. However, patients with ocular sequelae present significant permanent discomfort impacting their quality of life and high ocular morbidity requiring adapted management [8].

We conducted a national audit of ophthalmologic management practices during the chronic phase of EN in the 11 sites of the French reference center for toxic bullous dermatoses and severe drug reactions (TOXIBUL). We then compared our results with the literature. Based on this audit, we were able to propose an evaluation form to facilitate ophthalmic data collection during the chronic phase of EN as well as an algorithm for the ophthalmologic management of ocular sequelae of EN.

## Material and methods

First, we surveyed ophthalmologists and dermatologists at the 11 centers of the TOXIBUL reference center. We sent them a standardized questionnaire by email about care practices for the ocular sequelae of EN. The survey focused on the presence of a referent ophthalmologist at the center, the use of topical treatments (artificial tears, corticosteroid eye drops, antibiotic-corticosteroids, antiseptics, vitamin A ointment (VA), cyclosporine, tacrolimus), the management of trichiatic eyelashes, meibomian gland dysfunction, symblepharons, and corneal neovascularization, as well as the contactologic solutions implemented. If a center had more than one referent ophthalmologist, each one could answer independently of the others.

In a second step, we performed a literature review. We searched PubMed for all articles published between 1987 and 2021 dealing with the ophthalmologic management of the chronic phase of SJS/TEN. The bibliography search was guided by four themes: topical treatments, systemic immunosuppressive treatments, eyelid treatments, and adjuvant treatments (amniotic membrane transplantation (AMT), contactology, subconjunctival injection of anti-vascular endothelial growth factor VEGF). We selected the most relevant meta-analyses, cohort studies and retrospective case series according to the research themes for each treatment category. We excluded single case reports and articles on ocular management during the acute phase.

Finally, bringing together the results of the survey and data from the literature, we proposed an evaluation form to facilitate ophthalmic data collection during the chronic phase of EN as well as an algorithm for the ophthalmologic management of ocular sequelae of EN.

## Results of the survey

Eleven ophthalmologists and 9 dermatologists from 9 of the 11 centers completed the questionnaire (Table 1). All the dermatologists answered that they had a referent ophthalmologist for this disease at their center (9/9). The majority of ophthalmologists responded that they almost systematically prescribed preservative-free artificial tears (10/11, 91%) and VA (11/11, 100%), and, as needed, antiseptic or antibiotic eye drops (8/11, 73%), or antibiotic-corticosteroids eye drops (7/11, 64%). In the case of chronic inflammation, topical cyclosporine was consistently proposed (11/11, 100%). Tacrolimus eye drops (4/11, 36%) and autologous serum eye drops (6/11, 55%) were also proposed as needed, whereas systemic immunosuppressants were never used (0/11).

**Table 1:** Audit of ophthalmologic management practices during the chronic phase of epidermal necrolysis: Response from 11 ophthalmologists

Treatments	Responses (n = 11)
Artificial tears	10 (91)
Systematic	7/10 (70)
Sometimes	3/10 (30)
Preservative-free	10/10 (100)
Vitamin A Ointment	11 (100)
Systematic	6/11 (55)
Sometimes	5/11 (45)
Antiseptic or antibiotic eye drops	8 (73)
Systematic	0/8 (0)
Sometimes	8/8 (100)
Antibiotic-corticosteroid eye drops	7 (64)
Systematic	0/7 (0)
Sometimes	7/7 (100)
Cyclosporine eye drops	11 (100)
Systematic	1/11 (9)
Sometimes	10/11 (91)
Tacrolimus eye drops	4 (36)
Systematic	0/4 (0)
Sometimes	4/4
Autologous serum eye drops 20%	6 (55)
Systematic	0/6 (0)
Sometimes	6/6 (100)
Systemic immunosuppressants	0
Removal of trichiatic eyelashes	
By the ophthalmologist only	10/11 (91)
By the patient	3/11 (27)
By ciliary electrolysis	5/11 (45)
By argon laser	7/11 (64)
Removal of symblepharons	8/10 (80)
Systematic	0
Depending on the context	10/10 (100)
If difficulty fitting SL	8/10 80
If severe eyelid malposition	9/10 (90)
+ AMT or OMT	7/10 (70)
Corneal ulcer	
Amniotic membrane transplant	9 (82)
Corneal transplant	6 (55)
In case of meibomian gland dysfunction	
Eyelid hygiene	11 (100)
Systematic	5/11 (45)
Sometimes	6/11 (55)
Local antibiotics	10/11 (91)
Systematic	0
Sometimes	10/10
General antibiotics	8/11 (73)
Systematic	0
Sometimes	8/8 (100)
Indication for scleral lenses	
For disabling keratoconjunctivitis	10/10 (100)
For functional discomfort + visual impact	9/10 (90)
Anti-VEGF (corneal neovascularization)	7/11 (64)
Subconjunctival	6/7 (86)
Amniotic membrane transplant	0/7 (0)
Eye drops	2/7 (29)

*AMT* Amniotic membrane transplant, *OMT* Oral mucosa transplant, *SL* scleral lenses, *VEGF* vascular endothelial growth factor

The removal of trichiatic eyelashes was mainly performed by the ophthalmologist (10/11, 91%), rarely by the patient (3/11, 27%). The surgical release of symblepharon (i.e. a cicatricial fusion between the bulbar and tarsal conjunctiva) was not systematic (8/10, 80%), it was reserved in case of difficulty with contactology fitting (SL, 8/10, 80%) or of severe eyelid malposition (9/10, 90%), and was associated with AMT or oral mucosa transplant (OMT) to limit symblepharon recurrence (7/10, 70%).

In case of chronic corneal ulcers, most ophthalmologists recommended AMT (9/11, 82%) and some recommended tectonic keratoplasty (6/11, 55%) in case of large corneal perforation. In case of meibomian gland dysfunction, eyelid hygiene was recommended by all ophthalmologists (11/11, 100%) with specific topical antibiotics if necessary (10/11, 91%). All the centers proposed scleral lenses (SL) in the event of disabling keratoconjunctivitis sicca with functional discomfort and/or visual impairment (9/10, 90%). More than half of the centers used anti-VEGF in case of corneal neovascularization (7/11, 64%).

## Literature review

We retrieved 39 articles; 19 are presented in Table 2. The majority of the studies were retrospective and quite small in size. Few studies have assessed visual acuity in EN in the literature [5, 9].

**Table 2:** Literature review of the ophthalmologic management of ocular sequelae in SJS/TEN

Treatments	Author/(Ref)	Year	Methodology of the study	Number of patients	Conclusion
Local treatments					
Artificial tears	Saeed HN et al. [3]	2016	Literature review	Unknown	Instillation of preservative-free artificial tears is necessary and recommended to increase their volume while preserving the ocular surface
Vitamin A Ointment (VA)	Soong HK et al. [16]	1988	Multicentric randomized controlled	116 (EN = 24)	Study of the effect of (VA) (0.01%) versus placebo in patients with scarring conjunctivitis. Significant regression of conjunctival keratinization after application of VA. However, clinical symptoms and signs did not show significant improvement with the active drug compared to placebo
Corticosteroid eye drops	Kohanim S et al. [17]	2016	Meta-analysis	Unknown	Not recommended for long-term use; limited data, known harmful side effects. Short-term anti-inflammatory
	Prabhasawat P et al. [18]	2013	Prospective interventional comparative	30	Analysis of data from 30 patients with EN complicated by severe dry eye syndrome and treated with cyclosporin 0.05% 2 times a day for 6 months. Evaluation of dry eye symptoms, redness, break up time, fluorescein examination and Schirmer test before and after treatment (2, 4 and 6 months) Cyclosporine 0.05% eye drops may be beneficial in the treatment of chronic dry eye associated with SJS. 8 were excluded for poor tolerance and 5 were lost to follow-up. The remaining 17 cases all showed significant improvement in dry eye symptoms, conjunctival injection, superficial punctate keratitis (SPK), Schirmer's test (p < 0.05). Poor tolerance was manifested by pain, palpebral edema, ocular redness which could lead to discontinuation of treatment
	Wan KH et al. [19]	2015	Meta-analysis	1367	12 randomized controlled trials were analyzed to assess the efficacy of topical cyclosporin 0.05% treatment compared to a control group in dry eye syndromes (all causes including SJS). Compared to controls, patients receiving cyclosporine had significantly lower Ocular Surface Disease Index (OSDI) scores (p = 0.04), longer break up time (p = 0.04), improved Schirmer's score (p < 0.0001), reduced corneal fluorescein uptake (p = 0.03), and higher ocular surface red blood cell density (p = 0.004)
Antibiotics/antiseptics eye drops	Kittipibul T et al. [21]	2020	Prospective comparative study	40	A significantly higher proportion of various pathogenic microorganisms (mainly Pseudomonas aeruginosa, Staphylococcus aureus, Streptococcus and Acinetobacter) was found in EN (60%, vs. 10% in controls, p = 0.001) The use of local antibiotics must be appropriate and must be discussed on a case-by-case basis
Autologous serum 20% eye drops	Poon AC et al. [22]	2001	Prospective comparative clinical pilot study	26 eyes of 22 patients	Autologous serum was used in 15 eyes of 13 patients with resistant epithelial defects and in 11 eyes of nine patients with keratoconjunctivitis. The beneficial effect may be related to a number of active factors in the serum, including growth factors, fibronectin, vitamin A, and anti-proteases. In vitro toxicity testing showed that serum drops reduced toxicity compared with preservative-free eye drops. Their use in routine practice remains difficult
Topical tacrolimus	Lee YJ et al. [24]	2013	Retrospective consecutive case series	Unknown	Topical corticosteroids were left on continuously to control persistent and recurrent inflammation in SJS despite the introduction of tacrolimus
Systemic treatments					
(cyclosporine, azathioprine, cyclophosphamide, methotrexate, mycophenolate, dapsone andinfliximab)	Saeed HN et al. [3] Kohanim S et al. [17]				Systemic immunosuppression should be considered in cases of recurrent or persistent inflammation or perioperatively to control inflammation in preparation for and after ocular surgery
Eyelid treatments					
Oral mucosa transplant (OMT)	Osaki TH et al. [25]	2018	Meta-analysis	44 patients 63 eyelid (EN = 40)	The use of oral mucosa as a posterior flap transplant showed good functional and cosmetic results, long-term stability, and a low recurrence rate in the treatment of severe scarring entropion of the upper eyelid. Retrospective chart review of patients who underwent tarsotomy combined with OMT to treat severe upper eyelid scarring entropion. The primary underlying diagnosis was SJS (63%). Complete resolution (restoration of the upper eyelid margin to normal anatomic position with good cosmetic appearance) was achieved in 52 lids (83%). Recurrence occurred in 7 eyelids (11%)
	Fu Y et al. [28]	2011	Retrospective, interventional case series	22 (EN = 10)	Improvement in visual acuity in 13 eyes (59.1%) of patients after eyelid surgery
Amniotic Membrane Transplantation (AMT)	Kheirkhah A et al. (26)	2013	Retrospective study		In the case of severe symblepharon, an approach combining scar lysis, mitomycin application, OMT associated with sutureless AMT was a safe and effective technique for fornix reconstruction. In no case is AMT a substitute for OMT
Adjuvant treatments					
Subconjunctival anti- VEGF	Gueudry J et al. [31]	2010	Prospective study	13 eyes of 12 patients	Evaluation of the efficacy and tolerance of subconjunctival anti VEGF in 13 eyes. The percentage of corneal neovascularization to total corneal area decreased from 41.1 to 33.7% at day 45 (p = 0.0003) after an average of 2 to 4 injections. Visual acuity was not improved. Subconjunctival anti-VEGF injections decreased corneal neovascularization, with no significant improvement in visual acuity at 4 months. (All cases of corneal neovascularization)
Scleral lenses	Tougeron-Brousseau B et al. [9]	2009	Retrospective study	53 eyes of 42 patients	The use of scleral lenses was effective and safe for visual rehabilitation. A progression in visual acuity from 0.73 to 0.50 log (p = 0.0001) 6 months after scleral lens placement was shown. The mean Ocular Surface Disease Index (OSDI) improved from 76.9 22.8 to 37.1 26.7 (p = 0.0001). All patients included had a history of EN
	Sotozono C et al. [32]	2014	Retrospective study	94 eyes	Evaluation of the therapeutic benefits of scleral lenses in patients with ocular sequelae associated with EN. SL are safe and effective in improving vision and quality of life in EN patients with severe ocular sequelae
Limbal stem cell transplant	Venugopal R et al. [26]	2021	Prospective study	41 patients	Evaluation of Cultivated oral mucosal epithelial transplantation (COMET) in 41 patients with chronic EN sequelae. The evolution of corrected visual acuity, severity scores of various ocular surface parameters and the occurrence of complications were documented during a 2-year follow-up period. 82% of eyes (37/45) improved in visual acuity, 13% (6/45) had no change, while 2 eyes (4%) worsened in visual acuity. Two eyes developed persistent epithelial defects, with progression to corneal melting requiring keratoplasty
Amniotic Membrane Transplantation (AMT)	Yang Y et al. [35]	2021	Meta-analysis	41 patients	The amniotic membrane is used after the removal of the symblepharon. However, in end-stage EN, the effect of AMT is limited in corneal ulcers. Its effect is mostly recognized in the acute phase
Keratoplasty Penetrating keratoplasty (PK)	Wang F et al. [36]	2014	Retrospective study	10 eyes	Study of 10 SJS eyes. Penetrating keratoplasty was reserved for certain cases of corneal perforation, associating a patch graft with a conjunctival covering. Visual acuity improved in six eyes (60%), remained unchanged in three eyes (30%) and decreased in one eye (10%)
Osteodontokerato-prosthesis OOKP	Tan A et al. [38]	2012	Meta-analysis	96 patients	Main indications: EN and severe burns (96 EN). Anatomical survival in all OOKP studies was excellent, with a survival rate of over 80%, even after 20 years. Most frequent complications were: Glaucoma (47.2%) with difficulties in follow-up/management and postoperative vitreous hemorrhage but resolved within the first postoperative week in general. Need for specialized centers

19 articles were selected to best meet the interest of each treatment. 1 to 2 articles per treatment are represented in the table

### Local treatments

#### Artificial tears

Severe dry eye, the most common ocular sequelae, is associated with chronic inflammation of the ocular surface and is explained by tear deficiency, decreased corneal wettability, and increased tear evaporation [8]. The instillation of preservative-free artificial tears is necessary and recommended to increase their volume while preserving the ocular surface [3, 10-12]. Instillation of eye drops containing non-steroidal anti-inflammatory drugs is contraindicated as they are known to worsen corneal damage (ulceration, keratitis) that can lead to corneal perforation [13, 14]. Artificial tears should be used as required.

#### Vitamin A Ointment

VA ointment maintains the wettability of the ocular surface and limits conjunctival keratinization in patients with SJS/TEN [15] [16]. It should be used as required.

#### Corticosteroid eye drops

Due to their anti-inflammatory properties, short courses of topical corticosteroids reduce inflammatory ocular symptoms (redness, pain, burning) in patients. However, regular monitoring must be carried out to detect adverse effects such as infectious keratitis and elevation of intraocular pressure responsible for corticosteroid-induced glaucoma, and cataract [3, 10, 17]. These complications explain why long-term use of corticosteroids is not recommended. There is no consensus on the posology of eye drops. It depends on the presence and degree of clinical inflammation.

#### Cyclosporine eye drops

The beneficial effect of cyclosporine eye drops is recognized in the severe dry eye in EN allowing corticosteroid sparing and long-term instillation [18, 19]. The main problem with cyclosporine ophthalmic solution is its safety. Although one study showed a good safety profile over for 3 years [20], cyclosporine can be poorly tolerated with burning prickling, and foreign body sensation, all of which are reversible on discontinuation of treatment. Cyclosporine 0.05% is usually administered.

#### Antibiotic eye drops

A significantly higher proportion of various pathogenic microorganisms was found in conjunctival swabs in patients with EN (60% vs 10% in controls, p = 0.001) [21]. The use of local antibiotics must be appropriate, temporary, and discussed on a case-by-case basis as soon an infection is detected by bacteriological samples [8]. Antiseptics can be used for preventive purposes.

#### Autologous serum eye drops 20%

Autologous serum has the particularity of containing the elements present in the tear film allowing the regeneration and the proliferation of the epithelial cells of the cornea and the conjunctiva. Autologous serum contains essential components for the ocular surface such as vitamin A, fibronectin, epidermal growth factor (EGF), transforming growth factor-β (TGF-β) which allow the renewal of the ocular surface epithelium. Autologous serum also has an anti-inflammatory property through the action of interleukin-1 receptor antagonists, which explains its use in these diseases. Therefore, autologous serum eye drops could be useful in the treatment of ocular surface disease with cell damage. Their effectiveness has been demonstrated in the treatment of dry eye syndrome or in the persistence of corneal epithelial defects [10, 14]. In vitro toxicity tests showed that autologous serum eyedrops reduced toxicity compared to hypromellose, a substance forming the texture of eyedrops, present in preservative-free eye drops [22].

Indeed, the regulations and the manufacturing conditions of autologous serum eye drop require a blood transfusion center, which limits their use in practice. They are produced by centrifugation of the patient’s peripheral blood and then diluted in saline solution or artificial tears. Moreover, a risk of infection by contamination of eye drop bottles is not rare. A series of cases in Thailand evaluated a rate of 6.12% of positive cultures, including fungus, without clinical expression in patients [23]. The period of use varies in the literature from 1 day to 7 months, usually 4 times a day.

#### Topical tacrolimus

The role of tacrolimus in controlling ocular surface inflammation and reducing the use of local corticosteroids has been demonstrated in the literature[24]. In six patients with EN, the administration of tacrolimus ointment 0,02% in combination with local corticosteroid tapering therapy suppressed the inflammatory relapse. The corticosteroid sparing effect of tacrolimus thus made it possible to reduce corticosteroid-induced intraocular pressure by significantly reducing the need for local corticosteroids (p = 0.004). However, a complete cessation of corticosteroids treatment with topical tacrolimus was impossible because the inflammation of the ocular surface increased after discontinuation. A low dose of topical corticosteroids had to be maintained to avoid this inflammatory relapse[24]. Tacrolimus 0.02% ointment was topically applied 1 to 3 times per day, depending on disease severity, for up to 31 months.

### Systemic treatments

Some studies agreed that systemic immunosuppressants (cyclosporine, azathioprine, cyclophosphamide, methotrexate, mycophenolate, dapsone, and infliximab) should not be used except before ocular surgery to control inflammation to prevent inflammatory rebound or in cases of recurrent inflammation or moderate to severe involvement [3, 17]. However, the side effects of systemic immunosuppressants are not insignificant.

### Eyelid treatments

Examination of the eyelids is essential to identify eyelid malposition that may aggravate trauma to the ocular surface. The permeability of the lacrimal puncta, the position of the eyelashes, the state of the meibomian glands, the height of the lacrimal meniscus, the quality of the tear film, the depth of the fornices, and the presence of symblepharon(s), as well as the presence or absence of keratinization of the eyelid margin and the ocular surface are all elements to be taken into account [17].

Surgical management of trichiasis is common. Other anatomical abnormalities such as scarring entropion are possible requiring an OMT at the posterior lamella of the eyelid. This transplant provides good functional and aesthetic results as well as long-term stability with a low recurrence rate [25].

#### Oral mucosa transplant (OMT)

The use of the oral mucosa as a posterior flap transplant (marginoplasty technique) showed good functional and cosmetic results, long-term stability, and a low recurrence rate in the treatment of severe scarring entropion of the upper eyelid [25, 26].

AMT can also be used in combination with OMT or as an alternative for the repair of the fornix following the removal of the symblepharon. However, AMT should never be used instead of OMT when palpebral rigidity is required [27, 28].

#### Electrolysis treatment of trichiatic eyelashes

Several techniques have been compared in the literature to reduce the delay of eyelash regrowth using epilation, electrolysis, cryotherapy, or thermal ablation with argon laser [29]. In this experimental study comparing four rabbits, Argon laser thermal ablation was found to be an appropriate alternative to electrolysis or cryotherapy in some cases of trichiasis. Cryotherapy could promote the formation of symblepharons.

### Adjuvant treatments

#### Subconjunctival injection of anti-VEGF

Ranibizumab 0.1 ml injected subconjunctivally significantly inhibited corneal neovascularization as early as one-week post-injection in an experimental study comparing two groups of rabbits, one group receiving anti-VEGF and one control group (p = 0.001). No side effects were noted [30].

A prospective study of 12 patients conducted by Gueudry et al. in 2010 also showed regression of corneal neovascularization at day 45 after subconjunctival injections of anti-VEGF (bevacizumab 0.1 ml) (p = 0.0003) without improvement of visual acuity [31]

#### Scleral lenses

With their high oxygen permeability lens characteristics and non-contact geometry on the corneal surface, SL maintain a tear reservoir between the cornea and the posterior surface of the lens, thereby reducing patient discomfort and improving visual acuity by smoothing out corneal surface irregularities. A recent study demonstrated the therapeutic benefits of SL in the management of disabling ocular sequelae of EN [9]. Another study showed a significant improvement in visual acuity and quality of life after wearing SL [32]. Scleral lenses require a rigorous training of the patient for their daily manipulation. It is important to specify that SL can be difficult to fit in case of symblepharons and a reduced size of the fornix, hence the importance of fitting them in specialized centers [32].

#### Limbal stem cell transplant (LST)

Patients with limbal epithelial deficiency (such as EN) could be treated with autologous limbal transplantation [33]. However, this is only possible when there is enough limbus left and ocular damage is mostly bilateral in EN. Moreover, the residual limbus must be preserved on the valid eye in order not to cause a deficiency following harvesting since 70 to 80° of the limbal arc must be harvested to be effective.

Therefore, given the constraints and reduced success rate of limbal transplantation, the clinical use of epithelia obtained by ex vivo culture of autologous limbal stem cells could be considered [34].

Since allograft limbal transplants are subject to rejection, if both eyes are affected, the use of epithelial cells from the patient's oral mucosa (by removing the mucosa under local anesthesia) would allow the reconstitution of an epithelial layer in vitro within three weeks. A recent prospective study evaluating this technique of culture and grafting showed favorable results with an improvement of visual acuity at 2 years [26].

Few complications have been described, such as the risk of rejection and infection. The monitoring of postoperative healing is extremely important and requires an experienced team. Therefore, this technique is generally reserved for patients with good quality oral mucosa, which may be rare in EN, and to prepare the cornea for possible keratoplasty [34].

#### Amniotic membrane transplantation

In addition to its role in the acute phase of EN, AMT is known to be used in the management of chronic ulcers or after the removal of symblepharons, acting as a substrate for epithelial cells. However, the effect of AMT is limited in the severe sequelae of EN [8]. A recent study reported that patients who received acute AMT remained susceptible to chronic ocular damage and required close monitoring in the short, medium, and long term [35].

#### Keratoplasty

Transplantation, penetrating keratoplasty (PK), is reserved for certain cases of corneal perforation and associates a patch graft with a conjunctival flap. [36]. PK can be considered in EN when there is adequate limbal stem cell reserve and aqueous tear production and minimal or nonexistent eyelid margin and conjunctival keratinization. In the other cases, an osteodontokeratoprosthesis (OOKP) may be proposed. Although described more than 40 years ago, OOKP remains the keratoprosthesis of choice for end-stage corneal blindness that is not amenable to conventional PK. It is particularly resistant to a hostile environment such as the dry and keratinized eye resulting from severe EN.

The principle of this surgery is to use a tooth and its periodontium (periosteum—alveolar bone—alveolar-dental ligament) as biological support known as an "osteo-dental blade" for a synthesis optic of polymethylmethacrylate, thus constituting the OOKP, sutured to the cornea. This OOKP, once sutured to the cornea, is covered by a flap of jugal mucosa ensuring biological coverage of the device. The OOKP thus plays the role of the cornea allowing the light influx to be conducted to the retina.

This surgery requires cooperation with trained multidisciplinary teams (ophthalmologists, ENT or maxillofacial surgeon). Life-long follow-up is provided throughout life to detect and treat complications, which include oral, oculoplastic, glaucomatous, vitreoretinal and device extrusion complications [37]. Glaucomatous complications remain the most frequent postoperative complications inducing a decrease in visual acuity [38].

When surgery is decided a combination of limbal allograft, AMT, and tarsorrhaphy, followed by the use of serum-derived tears, could be proposed for ocular surface reconstruction [39].

## Conclusion

**Fig. 1:**
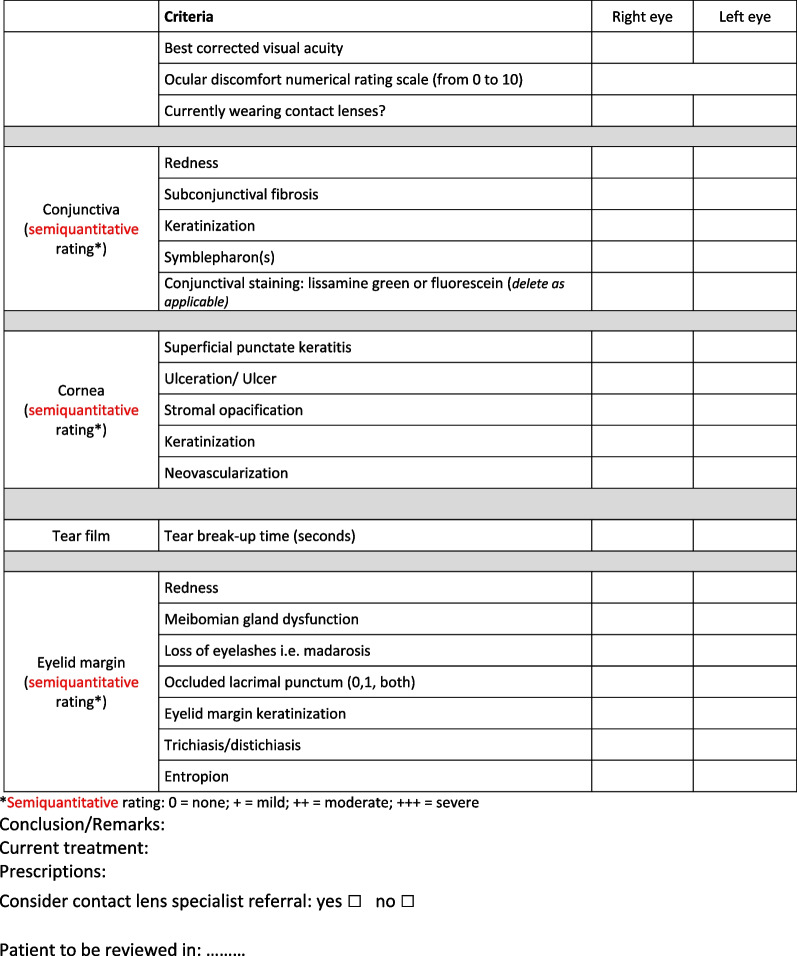
Ophthalmologic evaluation form in the chronic phase of epidermal necrolysis

**Fig. 2:**
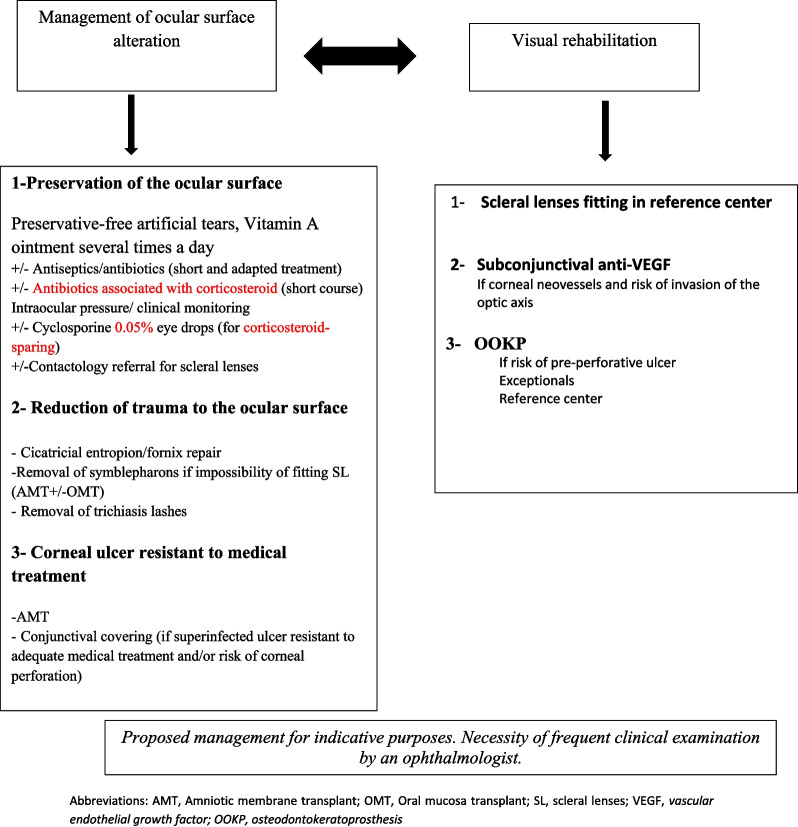
Algorithm for the ophthalmologic management of ocular sequelae during the chronic phase of epidermal necrolysis

Although there are no recommendations for the management of ocular sequelae in SJS-TEN syndromes, a certain uniformity of management in France has been demonstrated. Based on this practice audit and literature review, we propose an evaluation form to facilitate ophthalmic data collection in the chronic phase of EN (Fig. 1). We also propose an algorithm for the ophthalmologic management of ocular sequelae (Fig. 2), guided initially by the management of ocular surface alteration then, in a second step, we focus on visual rehabilitation.

## Data Availability

The data underlying this article will be shared on reasonable request to the corresponding author.
